# Corporate medicine is usurping the doctor-patient relationship in America

**DOI:** 10.1186/1878-5085-5-S1-A107

**Published:** 2014-01-28

**Authors:** Russell J Andrews

**Affiliations:** 1 Neurosurgery, Los Gatos, California, USA

## 

Not since the time of Hippocrates has the importance of the doctor-patient relationship been questioned. However, in the US a multitude of special interest groups - who benefit financially from the current system - continue to lobby for dismantling the long-term doctor-patient relationship in favor of a corporate model for health care delivery. More than even the teacher or the religious leader, the doctor bears a responsibility that transcends pure financial gain.

To understand how corporate, profit-driven medicine has evolved, one begins with the student entering medical school. Most incoming medical students are motivated to help others, but they quickly learn the consequences of putting the patient before the profit. In an academic position, one must bring in sufficient revenue to the university medical center or lose one's appointment; in private practice, one must be successful at both revenue generation and collection (dealing with dozens or more different insurance companies) or go bankrupt. The patient's health and welfare is virtually irrelevant to the doctor's economic survival.

Doctors in the US are being bombarded by corporate influences on their relationship with their patients. Television, the internet and other media are increasingly used for direct-to-patient marketing of expensive new prescription drugs, devices, and surgical procedures. Poorly-informed patients may seek the doctor who is willing to meet their demands rather than the doctor who takes the time to educate the patient on the most effective treatment. Moreover, since health care insurance companies have no guarantee their insured patients will continue with their company, there is no incentive for doctors to pursue preventive care.

Corporate medicine is replacing the primary "family doctor" - who follows the patient from infancy through adult life, both in and out of the hospital - with "doc-in-the-box" outpatient clinics and teams of hospitalist physicians. Both quality of care and cost of care suffer from this lack of continuity. A parallel development on both sides of the Atlantic has been limitation on the hours that physicians-in-training may work (both daily and weekly). Interestingly, the greatest "push-back" against these work hour limitations in the US has been from those in specialty training (e.g., neurosurgery) - the very people who it was argued are the most overworked. Neurosurgeons-in-training realize that neurosurgical disorders and their treatment do not adhere to a 9-to-5 schedule; these salaried physicians appear truly motivated to learn to provide the best care for their patients.

Physician medical societies in the US have transformed their primary role over the past decade from continuing medical education to continuing financial education. From the American Medical Association (AMA) to state specialty medical societies (e.g., CANS - the California Association of Neurological Surgeons) to local physician organizations (e.g., SJSS - the San Jose (California) Surgical Society), political action committees (PACs) have been formed to solicit contributions from their physician members to support lobbyists in Washington DC and state capitols, and consultants have been hired who can advise their physician members on how to maximize their personal financial gain.

Until doctors and patients demand major overhaul of the US health care system, the doctor-patient relationship - and the health of the entire country - will continue to suffer.

